# Cross-sectional study of Schmallenberg virus seroprevalence in wild ruminants in Poland at the end of the vector season of 2013

**DOI:** 10.1186/s12917-014-0307-3

**Published:** 2014-12-21

**Authors:** Magdalena Larska, Michał K Krzysiak, Julia Kęsik-Maliszewska, Jerzy Rola

**Affiliations:** Department of Virology, National Veterinary Research Institute, Al. Partyzantów 57, 24-100 Puławy, Poland; European Bison Breeding Center, Bialowieża National Park, Park Pałacowy 11, 17-230 Białowieża, Poland

**Keywords:** Schmallenberg virus, Wild ruminants, Seroprevalence, Risk factors

## Abstract

**Background:**

In view of recurrent Schmallenberg virus (SBV) infections all over Europe between 2011 and 2013, a lively scientific debate over the importance of the sylvatic transmission cycle of the virus has emerged. The study presents results of serosurvey which included wild ruminants representing species of red deer (*Cervus elaphus*), roe deer (*Capreolus capreolus*), European bison (*Bison bonasus*), fallow deer (*Dama dama),* mouflon (*Ovis orientalis musimon*) hunted or immobilized at 34 different locations of Poland in the autumn/winter 2013.

**Results:**

Out of 580 sera, 145 (25%) were considered positive for SBV antibodies. The overall SBV seroprevalence calculated using district probability weights was estimated at 27.7% (95% CI: 24.0-31.4). The seroprevalences at the district level varied between 0 and 80.0% (95% CI: 24.5-135.0%) with the mean within-district prevalence of 24.0% (95% CI: 16.5-31.4). Significantly higher seroprevalence was observed in animals from the Eastern provinces (36.6%) compared to the Western provinces (22.8%). SBV infection impact varied significantly between different species (higher SBV seroprevalence in larger species such as European bison), population type (free-ranging; captive), age, body weight, percent of the district forest area, part of Poland, and the densities of wild and domestic ruminants at the district and province level. Using statistical multivariable logistic model, population type, age, part of Poland and domestic ruminant density were identified as the main risk factors for SBV infection in wild ruminants in Poland.

**Conclusions:**

SBV seroprevalence in wild ruminants, similarly to the epizootic situation in domestic ruminants in the country, varied significantly between districts and provinces. Association between SBV seropositivity, species, animal body weight and age group expressed by a higher prevalence in larger ruminants may be explained by more frequent exposure to midge-vector bites of the latter, however it might also be related to the different species susceptibility to SBV infection. The positive effect of higher domestic ruminant density on the risk of SBV infection in wildlife and lower SBV seroprevalences in the latter suggested that the sylvatic cycle of SBV transmission is an effect of the pathogen spillover from the domestic animals.

## Background

Schmallenberg virus (SBV) is a novel *Orthobunyavirus* infecting ruminants which emerged in North Rhine-Westphalia, Germany in August/September 2011 [1]. SBV transmission occurs through midge vectors and infection is mostly sub-clinical in adult animals [1]. The virus infections in pregnant domestic ruminants were observed to provoke abortions, stillbirths and, most frequently, congenital musculoskeletal and neural malformations observed in new-born animals leading to their death shortly after birth [2]. In view of recurrent SBV infections all over Europe, lively scientific debate over the importance of the sylvatic transmission cycle of the virus has emerged [3]. SBV infection has been already reported in the wildlife in Poland [4]. This study presents wider perspective of SBV infections in wild ruminants in Poland.

## Methods

### Sample collection

**Figure 1:**
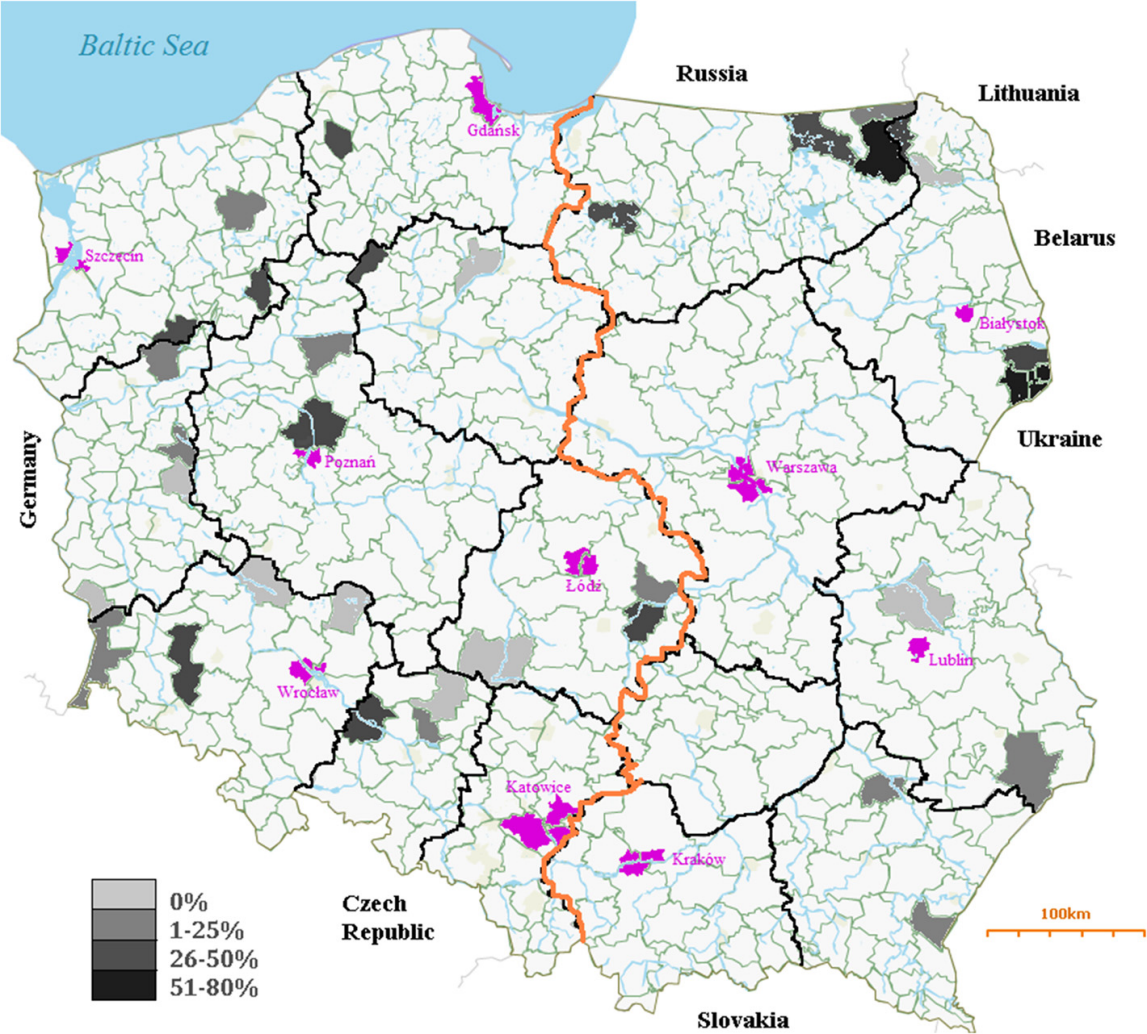
**Seroprevalence of SBV according to the location.** The forest districts were marked with different shades of grey corresponding to district population weighted SBV seroprevalences (legend). The orange line divides the parts of Poland (Western on the left, Eastern – on the right). The main cities are marked in purple.

A total of 580 serum samples collected during collective hunting or from immobilized, selectively culled or fallen red deer (*Cervus elaphus*) (n = 176), roe deer (*Capreolus capreolus*) (n = 66), European bison (*Bison bonasus*) (n = 11), fallow deer (*Dama dama*) (n = 256), and mouflon (*Ovis orientalis musimon*) (n = 71) were tested. The samples were collected during the hunting season 2013/2014 (from 25 October 2013 until 29 January 2014) from animals originating from 34 forest districts (23 located on the Western side of Vistula river, assigned as Western Poland in the statistical analysis). The study was based on a cross-sectional design aiming to estimate the prevalences of infections with BVDV and BoHV-1. The density of wild ruminant population and species composition according to the information provided by the National Forest Holding [5] and published by Central Statistical Office [6] were considered when selecting the distribution of sampling sites and the number of animals. The average number of animals sampled was 17.1 (95% CI: 3.5-30.7) per district. The species were homogenously distributed per district, except for European bison and mouflon which originated from two districts with the largest population sizes in Białowieża Primeval Forest (East Poland, near Białystok) and Pławin (West Poland, near Szczecin) (Figure 1), respectively. Cervid samples were collected during collective hunting according to the appropriate Polish legislation [7], or in case of the farmed animals by mechanical immobilization. No ethical/welfare authority approval was required as samples were collected post-mortem or obtained at the request of the owner for diagnostic purposes from live animals immobilized by certified veterinarians. The European bison were sampled after chemical immobilization [8] or culling by certified hunters in accordance with the appropriate regulations [9]. The blood was collected into sterile tubes with clot activator and gel for serum separation from the external jugular vein, heart and body cavities from dead animals, while it was drawn from jugular vein from the immobilized ones. Sampling date, location, gender and body weights were recorded. More females (n = 307) than males (n = 133) were sampled, while the information of the gender was missing for 140 animals. The average age was 3.2 (95% CI: 2.8-3.7) years. The majority of samples (n = 539) derived from animals older than one year. The oldest ruminants tested were European bison with the average age of 12.6 (95% CI: 8.2-17.1) years. The body weight data was available for 345 animals with the average of 64.7 (95% CI 57.0-72.4) kg. The continuous values were transformed into ordinal variables for the statistical analysis. The body weights were associated with the animal species with the mean values of 17.4 (95% CI: 14.9-19.8), 23.7 (95% CI: 16.9-30.4), 55.4 (95% CI: 72.2-82.0), 77.1 (95% CI: 72.2-82.0) and 382 (95% CI: 82.2-681.7) kg for roe deer, mouflon, fallow deer, red deer and European bison, respectively.

### c-ELISA

Serological ID Screen Schmallenberg Virus Competition Multi-Species ELISA (ID.vet, France) for the detection of antibodies against SBV nucleoprotein was performed according to the manufacturer’s instruction. The results were interpreted on the S/P values calculated from the optical density (O.D.) values measured at 450 nm using the following equation: O.D. _sample tested_ – O.D. _negative control_ / O.D. _positive control_ – O.D. _negative control_ × 100. The S/P values below and equal to 40% were considered positive, S/P value above 50% - negative, and the S/P value between 40% and 50% doubtful.

### Virus neutralization test (VNT)

Randomly selected non hemolyzed serum samples giving negative (n = 20), positive (n = 50) and doubtful (n = 12) results by c-ELISA were verified by virus neutralization test. The sera were heat-inactivated at 56°C for 30 min beforehand. Serial two-fold dilutions of the sera (starting from 1:4 up to 1:4096) in Glasgow Minimum Essential Medium (GMEM) were incubated for 1–1.5 h at 37°C in a 96-well microplate with approx. 500 TCID_50_ of the Polish SBV strain 130/6 isolated from the cerebrum of ovine fetus. The sera were tested in duplicate. The strain was passaged 3 times in baby hamster kidney (BHK-21) cells with the final titer of 8 × 10^4^ TCID_50_. Back titration of virus was included in each assay by performing four 10-fold dilutions of the virus working solution. Two wells with 1:4 dilution of each serum were left uninfected as a serum cytotoxity control. Similarly diluted negative (commercial foetal bovine serum negative in ELISA) and positive (bovine sample from a confirmed SBV outbreak) serum samples were tested simultaneously. After the incubation, each well was overlaid with approx. 10^4^ BHK-21 cells and the plates were incubated for five days at 37°C in 5% CO_2_. After fixation in 80% chilled acetone, drying, staining with 0.1% crystal violet and washing, the plates were examined under the microscope. Serum was considered as a VNT positive if the viral CPE inhibition was observed in at least one of two wells at a dilution ≥1:4. The titer of tested serum sample was assumed as the reciprocal of the highest dilution of the sample for which none or one of the wells of a dilution showed any cytopathic effect.

### Statistics

Statistical analysis was performed using STATA v. 13.0 software (StataCorp LP, Texas, USA). The prevalences were corrected by the population probability weights (pweights) calculated as follows: 1/(n of sampled animals/N of animals in the forest district). The latter together with forest density data was obtained from the General Directorate of National Forest Holding [5]. The densities of livestock at the province level derived from yearbooks of Central Statistical Office [10]. The associations between the gender, age group, body weight, population type (free-ranging; captive), geographic location, part of the country (Western; Eastern), wild and domestic ruminant densities per km^2^ and SBV seroprevalence were estimated using χ2, Spearman test and odds ratio (OR) calculated by univariate logistic regression. The variables are explained in detail in Table 1. In order to asses the risk factors of SBV seropositivity, mixed-effects logistic regression model accounting for species as fixed effect was compiled using backward procedure. The district-level wild ruminant density and body weight group variables were removed from the model due to collinearity (Spearman coefficient ρ_S_ > 0.5; *p* < 0.05).

**Table 1:** **Schmallenberg virus seroprevalence in relation to different characteristics of the wild ruminants and their origin in the univariable analysis**

**Variable**	**Category (values)**	**SBV seroprevalence**			**χ2**	***P*** ^**4**^	**OR** ^**5**^	**OR 95% CI**
		**n/N** ^**1**^	**%** ^**2**^	**95% CI** ^**3**^				
Animal species	Fallow deer (*Dama dama*)	81/256	22.7	17.5-27.8	49.4	<0.001	0.8	0.7-1.0
	Red deer (*Cervus elaphus*)	44/176	30.6	23.7-37.5				
	Mouflon (*Ovis aries musimon*)	1/71	1.4	−1.4-4.3				
	Roe deer (*Capreolus capreolus*)	10/66	22.6	12.2-33.0				
	European bison (*Bison bonasus*)	9/11	81.8	54.6-109.0				
Population type	Free-living	63/289	22.1	17.2-26.9	3.1	0.08	1.4	1.0-2.0
	Captive	82/291	69.1	63.7-74.4				
Gender (n = 440)	Female	63/307	31.7	26.4-36.9	0.02	0.9	1.0	0.6-1.7
	Male	28/133	14.8	47.3-64.0				
Age group	Below and equal to one year of age	1/41	1.7	−2.5-59.3	12.0	0.001	14.6	2.0-107.0
	Over 1 year of age	144/539	28.6	24.8-32.4				
Body weight group (n = 345)	1 (mean 15.9; range 8–25)	10/72	19.8	10.4-29.3	32.9	<0.001	2.1	1.5-2.9
	2 (mean 40.2; range 30–50)	6/116	3.3	0.008-6.6				
	3 (mean 76.7; range 55–100)	30/126	26.1	18.3-33.8				
	4 (mean 188.6; range 110–650)	14/31	42.4	23.9-60.8				
Percent of the forest area in district	Low (mean 25.8; range 20.1-30.9)	10/61	17.4	7.6-27.2	8.8	0.01	1.8	1.2-2.8
	Medium (mean 40.8; range 35.2-66.0)	110/454	25.0	21.0-29.0				
	High (mean 85.9; range 67.5-97.3)	25/65	36.0	24.0-48.0				
District-level density of wild ruminants (animals per km^2^)	Low (mean 2.8; range 2.1-3.4)	3/13	17.4	−6.4-41.2	23.8	<0.001	0.4	0.3-0.66
	Medium (mean 5.5; range 4.1-6.3)	94/275	31.3	25.8-36.9				
	High (mean 8.3; range 6.7-8.7)	48/292	15.7	11.5-19.9				
Part of country	Western	72/434	22.8	18.9-26.8	65.0	<0.001	5.0	3.3-7.6
	Eastern	73/146	36.6	28.7-44.5				
Province-level density of domestic ruminants	Low (mean 5.5; range 4.2-14.0)	64/390	20.0	16.1-24.1	48.2	<0.001	2.7	2.0-3.6
	Medium (mean 21.1; range 17.7-28.5)	62/152	34.1	26.5-41.7				
	High (mean 44.4; range 44.4-44.4)	19/38	42.0	25.6-58.4				

^1^number of SBV seropositive samples / number of tested samples; ^2^seroprevalence adjusted for forest district population weights; ^3^confidence interval calculated by Wald test^; 4^p < 0.05 was considered significant; ^5^odds ratio.

## Results and discussion

**Figure 2:**
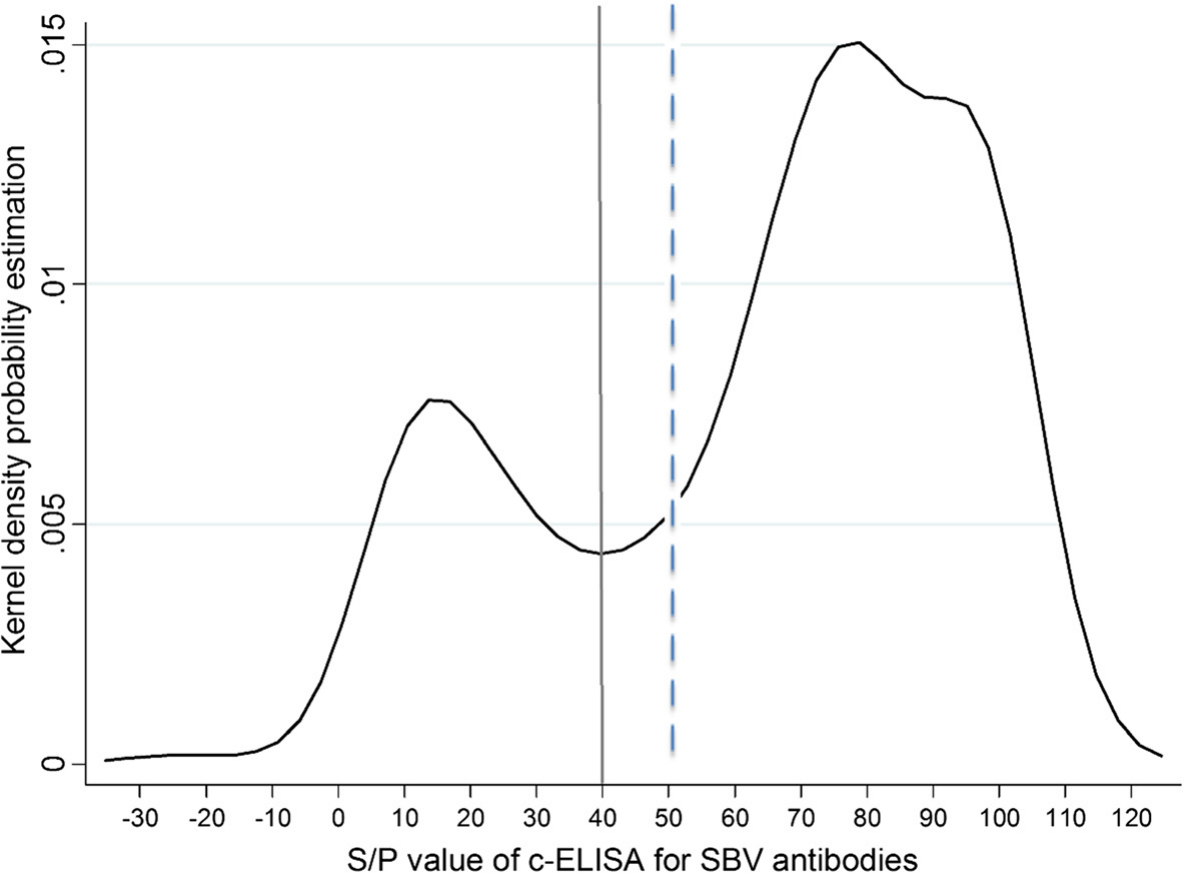
**Distribution of S/P values of ID Screen Schmallenberg Virus Competition Multi-Species ELISA (IDvet, France) for 580 serum samples collected from wild ruminants.** The vertical lines indicate the cut-off values. The results below and equal to 40% were considered positive, S/P value above 50% - negative, and the S/P value between 40% and 50% - doubtful.

Based on c-ELISA, of 580 serum samples 136 (23.4%), 29 (4.8%) and 415 (71.6%) were considered positive, doubtful and negative for the presence of SBV antibodies, respectively. The Kernel density plot showed clear bimodal ELISA S/P value distribution divided by the cut-off value (Figure 2). Some selected for their good quality, non hemolyzed sera were tested with VNT. The c-ELISA positive (n = 50) and negative (n = 20) results were confirmed consistent by VNT, while 9 (75%) and 3 (25%) out of 12 c-ELISA doubtful sera were VNT positive and negative, respectively. The mean VNT titers of c-ELISA positive and doubtful sera were 560.3 (95% CI: 324,7-796.0) and 210.3 (95% CI: −47.9-468.4), respectively. For further analysis, the number of SBV seropositive wild ruminants was adjusted according to VNT results at 145 (25%) animals, while the rest of the doubtful sera (n = 17) unsuitable for VNT due to their cytotoxity were considered negative. The problem of poor quality samples originating from wildlife and the usefulness of confirmatory tests was discussed in detail by Casaubon et al. [11] in a similar serosurvey of bluetongue virus in free-ranging cervids and wild *Caprine*. Another possible bias derives from the differences in ELISA and VNT sensitivities. In serology, VNT is considered as a gold standard and used as a confirmatory method to the commercial tests also in SBV epidemiology. The same c-ELISA (ID.vet) was successfully used in other wild ruminant studies before [12-14], however Laloy et al. [15] have pointed out the possible disagreements between the two methods. In their study, c-ELISA has failed to detect 30 out of 97 (30.1%) VNT positive results of red deer sera, while the false positive ratio reached 35.3%.

Using the forest district population weights, the overall SBV seroprevalence was estimated at 27.7% (95% CI: 24.0-31.4). The percentage of SBV seropositive wild ruminants was slightly lower than the apparent seroprevalences for wild ungulates observed at the beginning of the European epizootic in Belgium (43.1%) [16] and in UK (approx. 56% in fallow deer and 71% in red deer) in 2012 [12]. In Poland, after the first detection of SBV infection in domestic ruminants during summer of 2012 [17,18], SBV transmission to free-ranging red deer in Western and European bison in Eastern Poland were detected in November 2012 [4]. The seroprevalence in wild ruminants was comparable to the value of 35.6% observed in domestic ruminants in Poland in 2013 [19] which suggests similar exposure to SBV infection. It is consistent with the observation of the ecosystem of Spanish hunting estate where SBV seroprevalence in roe deer (80%) was almost identical to the cattle herds (86.8%) reared in the area [14].

The SBV seroprevalences of the 34 forest districts included in the study ranged between 0 and 80.0% (95% CI: 24.5-135.0%) with the mean within-district prevalence of 24.0% (95% CI: 16.5-31.4) (Figure 1). No seropositive wild ruminants were found in nine (26.5%) districts of which seven were located in Western Poland. Significant associations between SBV seroprevalence and environment conditions such as part of the country where the animals originated from, the percent of forest area in the district and wild and domestic ruminant densities were also found (Table 1). Higher proportion of SBV seropositive wild ruminants was found in Eastern Poland, in the districts with higher percent of forest area and in the provinces characterized by the highest densities of domestic ruminants. However, strong dependence between wild and domestic ruminant densities (ρ_S_ = −0.6; *p* < 0.0001), or their relation with the part of Poland (ρ_S_ = −0.5; *p <* 0.0001 and ρ_S_ = 0.6; *p <* 0.0001, respectively), or association between the part of country and the percent of forest area (ρ_S_ = 0.3; *p <* 0.0001) should be considered when interpreting those results. High geographic variation in SBV seroprevalence in wildlife was described also in France and Belgium [15,16]. The differences may be related to the vector exposure of wild host species in relation to their habitat, vector abundance or population structure. In the Spanish study, SBV infection rate of roe deer inhabiting the lowlands was significantly higher than the low prevalence in Pyrenean chamois and mouflon residing in the high mountains of the same national park [14]. Another factor determining the differences in SBV exposure may be connected to the species and size of the animal (age and body weight). In our study, SBV seroprevalence differed significantly between the species and body weight of wild ruminants, with European bison (81.9%) and the heaviest animals (42.4%) showing the highest seroprevalences (Table 1). It is consistent with the previous study which showed lower seroprevalences in smaller ruminants (sheep and goats) in respect to cattle [19]. At this point, the limitation of the study should also be considered. Table 1 presents the results of the univariable analysis, however some variables such as wild and domestic ruminant densities, part of Poland and forest area were cross-correlated and therefore might influence the interpretation of the results. Despite the assumption of homogenous distribution of the different species in the country, one should be aware that sampling of large wildlife species is restricted by local regulations or practices which might affect the interpretation of statistical analysis. Whether the differences in SBV seropositivity are connected to the species susceptibility, the animal size or age is difficult to determine with certainty, especially as those characteristics are closely related. The differences of species susceptibility to SBV infection have been studied experimentally. Poskin et al. [20] and Wernike et al. [21] have shown that a lower infectious dose of SBV is required to infect cattle in comparison to sheep which would need ten times higher dose of the virus to get seroconversion in all inoculated animals. Therefore the higher seroprevalence in bison may reflect their higher susceptibility as in cattle. Another explanation might be the higher production of carbon dioxide in the larger ruminant species which is one of the strongest midge attractants and may promote higher exposure to their bites and hence to SBV infection. Studies on the feeding behavior confirm the observation. According to Lassen et al. [22] and Ayllón et al. [23] among others, most *Culicoides* species feed preferentially on cattle. In relation to high SBV infection rate in European bison, the specific environment of the tested population should also be considered important. The European bison included in the study originated from a reserve at the Białowieża Forest which is known for their vast marshes and therefore abundant in the midge vectors. The area is neighboring with Polish Eastern borders. Interestingly, the SBV prevalence in wild ruminants was increased in the Eastern part of the country which is known for higher density of cattle reared extensively in smaller size herds, often pastured enabling SBV transmission at the interface between domestic and wild ruminant populations [10]. In contrast to Eastern Poland, the density of domestic ruminant population on the Western side of Vistula river is lower, while the population size of wild ungulates is significantly higher. Those observations suggest that SBV infection in wild ruminants is more likely to be the effect of spillover from domestic livestock and not the opposite. Probably, the environment characteristics such as humidity, temperature, variation in temperature during night and day affecting the presence and density of vector influence the possible transmission and consequently the prevalence in both domestic and wild animals. Perhaps the role of SBV wildlife reservoirs threatening domestic ruminants should not be overemphasized [3]. In case of endangered and protected species such as European bison, the threat to the reproduction and health of the animals should not be underestimated. A single clinical case connected to SBV infection have been already described in an elk calf in Poland, however the association could not be proven [4]. Species susceptibility should be investigated further.

In order to estimate the risk factors of SBV infection in wild ruminants in Poland, the associations between SBV seropositivity and animal-level variables were tested by multivariable mixed-effects logistic regression model. The final model which included four risk factors for SBV infection and species as fixed effect was developed for the wild ruminants in Poland (Table 2). The collinear variables were excluded. The odds of SBV seropositivity were significantly higher for the animals which were reared in captivity (OR = 3.2, *p* = 0.004) in respect to free-ranging animals. Different environment characteristics could influence the epidemiological role of the different species as well as the biological behavior of free ranging animals. Different land use of free ranging could increase or reduce the contact with vectors. And again, already discussed and related risk factors for SBV infection: the origin from the Eastern part of the country and the higher densities of domestic ruminant population were found significant in the model. Interestingly, as already observed in the univariable analysis, SBV seroprevalence was associated with animal age also in the final model. The risk of SBV infection was ten times higher in older than 1 year animals in comparison to the younger ones. First of all, this association may again suggest the association to the size of the animal, however young wild animals are always in direct contact with adults, therefore the body dimension should not explain the different seroprevalences. Notwithstanding, decrease of seropositive young animals indicating smaller exposure of animals born in 2013 may imply the subside of SBV epidemic in Poland. Additionally, passive immunity should be considered important when estimating the true prevalence in the youngest animals. Recent study of Elbers et al. [24] have shown the decay of maternal antibodies in calves 5–6 months after their birth, however the decline of passive immunity can vary depending individual species of wild ruminants and it may proceed at a different pace than in cattle. The animals below one year of age belonged to all the species included in the study and originated from five different hunting districts spread evenly across the country. Ten out of 41 youngest animals were equal or below 6 months of age. The single SBV seropositive one among them was 6-months-old European bison from Białowieża Primeval Forest, located at the extreme east of Poland.

**Table 2:** **Risk factors associated with SBV infection in wild ruminants in Poland in 2013 estimated by multivariable mixed-effects logistic regression model with species as fixed effect (** ***p*** **< 0.0001)**

**SBV seropositivity**	**OR**	**SE**	***z***	**P > |** ***z*** **|**	**OR 95% CI**
Population type					
free-ranging	1*	-	-	-	-
captive	3.2	1.3	2.9	0.004	1.4-7.2
Age group					
0-1 years	1	-	-	-	-
>1 year	10.4	11.2	2.2	0.03	1.2-86.4
Part of country					
Western	1	-	-	-	-
Eastern	2.2	0.7	2.6	0.009	1.2-4.1
Province-level density of domestic ruminants					
low	1	-	-	-	-
medium	2.3	0.7	2.8	0.005	1.2-4.1
high	1.6	0.8	2.6	0.4	0.6-4.4
Intercept	0.01	0.01	0.88	<0.001	0.001-0.1

*reference level; n = 580 observations.

## Conclusions

Wild ruminants might play a role in SBV transmission in Poland, however lower seroprevalence in relation to the domestic ruminants suggests the spill-over effect from the latter, rather than inverse. The higher SBV seroprevalence in the Eastern part of the country suggests eastbound transmission of SBV in Europe, while low SBV prevalence in the wild ruminants below one year old may indicate possible decline of SBV epidemic in Poland. The species and size of the ruminant seem to be significant risk factors for SBV infection, probably due to the different species susceptibility and/or increased attraction of midges to larger animals.
